# Risk of liver injury after α-glucosidase inhibitor therapy in advanced chronic kidney disease patients

**DOI:** 10.1038/srep18996

**Published:** 2016-01-11

**Authors:** Chih-Chin Kao, Pei-Chen Wu, Che-Hsiung Wu, Li-kwang Chen, Hsi-Hsien Chen, Mai-Szu Wu, Vin-Cent Wu

**Affiliations:** 1 Division of Nephrology, Department of Internal Medicine, Taipei Medical University Hospital, Taipei, Taiwan; 2 Graduate Institute of Clinical Medicine, College of Medicine, Taipei Medical University, Taipei, Taiwan; 3 Division of Nephrology, Department of Internal Medicine, Mackay Memorial Hospital, Taipei, Taiwan; 4 Division of Nephrology, Taipei Buddhist Tzu Chi General Hospital, Buddhist Tzu Chi University, Taipei, Taiwan; 5 Institute of Population Health Sciences, National Health Research Institutes, Zhunan, Taiwan; 6 Department of Internal Medicine, School of Medicine, Taipei Medical University, Taipei, Taiwan; 7 Department of Internal Medicine, National Taiwan University Hospital, Taipei, Taiwan; 8 National Taiwan University Study Group on ARF, (NSARF).

## Abstract

Although α-glucosidase inhibitors (AGIs) are commonly used for controlling postprandial blood glucose, AGIs-induced liver injuries have been reported. However, the relationship between AGIs and liver injuries in advanced chronic kidney disease (CKD) patients remains unexplored. In this nationwide case-control study, we recruited 1765 advanced diabetic CKD patients, who received AGIs therapy from January 1, 2000 to December 31, 2010 as the study sample and 5295 matched controls. Recent and former AGIs users were defined as patients who received the AGIs prescription for 30–60 d and 30–210 d before the event of liver injury. The risk of AGIs-induced liver injury was examined using time-dependent Cox proportional hazards model. Liver injury occurred in 3.9% of patients in the study group and 3.3% of patients in the control group. AGIs use did not increase the risk of liver injury in advanced CKD patients (*P* = 0.19). The stratified analysis indicated no increased risk of liver injury in all AGIs-using subgroups (all *P* > 0.05). The available evidence supports extending the use of AGIs without increasing the risk of liver injury in patients with advanced CKD. Additional randomized controlled trials are warranted to confirm our results.

The liver is the most vital organ for the metabolic disposition of all drugs, and therefore, drug-related liver injury is a potential complication of nearly every medication^1^. Alpha-glucosidase inhibitors (AGIs) are oral hypoglycemia agents (OHAs) that inhibit α-glucosidase in the brush border of the small intestine for delaying carbohydrate breakdown and reducing postprandial hyperglycemia. Several clinical trials have reported elevated liver enzyme levels in patients who received AGIs therapy compared with those who received a placebo^2^,^3^. Several case reports have already reported reversible hepatotoxicity after AGIs use^4^,^5^,^6^,^7^, along with re-exposure confirmation. The exact mechanism of AGIs-induced liver injury is idiosyncratic^8^. The peak plasma concentrations of patients with advanced chronic kidney disease (CKD) who received AGIs were approximately 5 times higher than those of volunteers with normal renal function^9^. Therefore, AGIs are not recommended for patients with advanced CKD because of the risk of accumulation and lack of studies that support this prescription. However, many advanced CKD patients receive AGIs for glucose control^10^ because AGIs have been shown to be effective, safe, and well tolerated in a large cohort of Asian patients with type 2 diabetes^11^; therefore, AGIs are of particular research interest. Many practitioners continue to prescribe AGIs even when the estimated glomerular filtration rate (eGFR) falls to less than 25 mL/min/1.73 m^2^, possibly with dose adjustments, to account for reduced renal clearance of the compounds^10^,^12^. A Taiwanese study reported that AGIs were used in 6.4% of CKD patients^13^. Oral AGIs are poorly absorbed from the gastrointestinal tract and then almost completely eliminated in the urine^14^, and renal failure greatly influences pharmacokinetics and drug plasma levels. However, the exact association between AGIs and the risk of liver injury in advanced CKD is not warranted, which makes it difficult to determine this association in end-stage renal disease (ESRD) patients. We examined the relationship between the use of AGIs and liver injury in advanced CKD patients in a Taiwanese population-based cohort.

## Results

This study included 1765 diabetic patients with advanced CKD who received AGIs therapy and 5295 diabetic patients with advanced CKD who did not receive AGIs therapy as matched controls. The characteristics of these 2 groups of patients are summarized in Table 1. The mean age of the enrollees was 63 ± 11 y and nearly 51% were men. The baseline comorbidities, including cardiovascular comorbidities, and the Charlson score, were comparable. The chronic hepatic comorbidities, including liver cirrhosis and alcoholic or viral liver diseases, did not differ between the groups. Patients in the study group were more likely to experience subsequent ESRD during the study period (92.3% vs. 82.5%). Insulin and OHA (including biguanide, dipeptidyl peptidase 4 (DPP4 inhibitors), meglitinide, thiazolidinedione, and sulfonylurea) were more frequently used in the study group (all *P* < 0.05).

### AGIs versus the risk of hepatic injury

AGIs prescription was not associated with an increased risk of liver injury in advanced CKD patients (hazard ratio [HR] 1.56, 95% confidence interval [CI] 0.80–3.04, *P* = 0.22) among recent users (HR 2.92, 95% CI 0.27–31.48, *P* = 0.38) or former users (HR 0.39, 95% CI 0.04–4.31, *P* = 0.44) while accounting for the cumulative time-varying measurements of the other OHA mediators of interest. The models revealed an increased risk of liver injury in men, and baseline liver disease patients. The C-index value (0.56) indicated high validity for the model (Table 2). The sensitivity analysis, which excluding baseline liver disease also proved that AGIs was not associated with increased risk of liver injury (supplement table 2).

**Figure 1:**
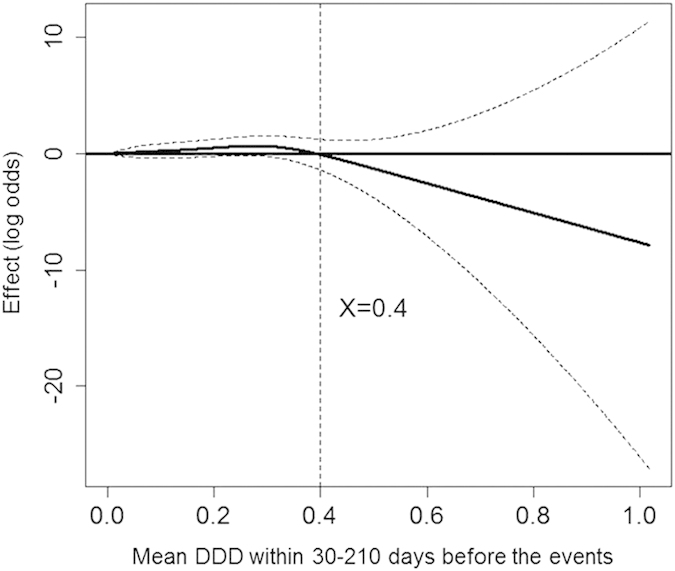
The mean DDD and risk of liver injury. The function curve with values of the logs of odds ratios from the GAM with splines regarding AGIs use for multilevel discrete-time event history analysis of the risk of liver injury among advanced chronic kidney disease patients of our study. The curve was centered to have an average of zero over the range of the data. The dashed lines indicated approximated point-wise 95% CIs. (Abbreviations: CI, confidence interval; DDD, defined daily dose; GAM, generalized additive model).

To evaluate the cumulated dose effect of AGIs on liver injury in type 2 diabetes mellitus patients, DDD within 30–210 d before the event was plotted on GAM plots (Fig. 1). A turning point is defined as the maximum slope of the tangent line on the basis of the curve. The mean DDD within 30–210 d (<0.4, which was equal to 120 mg of acarbose) was associated with a non-significant increased risk of liver injury, as indicated by the turning point.

**Figure 2:**
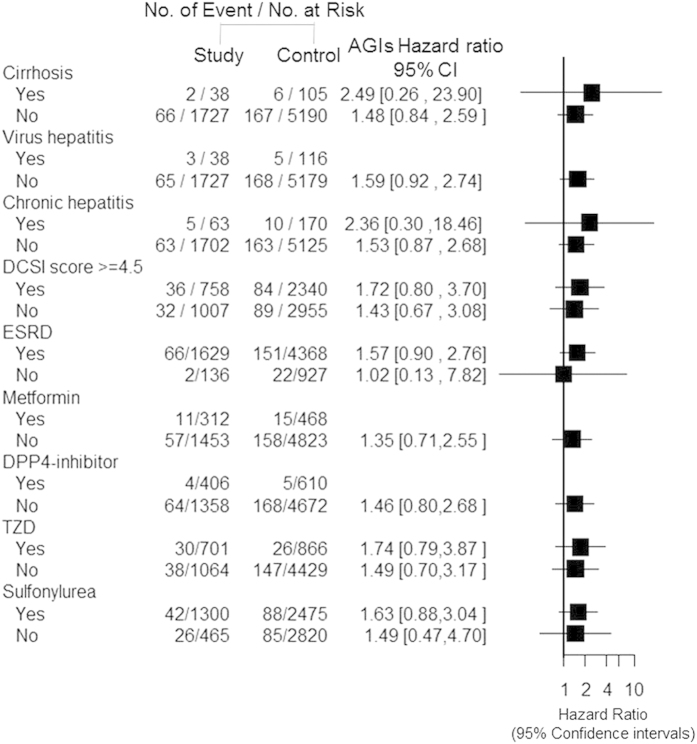
Multivariate stratified analysis indicated no increased risk of liver injury in all α-glucosidase inhibitor (AGIs)-using subgroups.

The multivariate stratified analysis revealed no increased risk of liver injury across all examined subgroups, including the presence or absence of liver cirrhosis, viral hepatitis, chronic hepatitis, subsequent ESRD, or each OHAs use, including metformin, DPP4-inhibitors, thiazolidinedione, or sulfonylurea (Fig. 2). Moreover, a high or low DCSI score based on diabetes-related organ damage was not associated with an increased risk of liver injury after AGIs use.

## Discussion

AGIs are not recommended for patients with creatinine clearance <25 mL/min/1.73 m^2^ because studies examining the safety of AGIs use in patients with advanced CKD are limited. In our population-based study, no increased risk of acute liver injury after AGIs use was observed in advanced CKD patients. Our results are consistent for recent and former AGIs users, and patients with chronic liver disease or those who eventually developed ESRD.

First approved in 1995, AGIs inhibit intestinal α-glucosidase, an enzyme for digestion and absorption of starch, disaccharides, and dextrin, and are commonly used for controlling postprandial blood glucose. In the gastrointestinal tract, 1–2% of oral AGIs are absorbed and then almost completely eliminated in the urine. AGIs have well-documented safety profiles^15^ and have been reported to effectively treat diabetes, with an advantage of reducing cardiovascular risk. Several studies have reported that the effects of AGIs as a monotherapy are similar to those of other antidiabetic agents in mitigating glycosylated hemoglobin and blood glucose levels, and the effects of AGIs are similar to those of metformin, as reported in a Chinese-based cohort^16^. AGIs use in advanced CKD patients is avoided because this therapy has not been studied in patients with severe renal impairment.

The incidence of AGIs-induced liver injury is considerably low and unpredictable^17^. In a phase III American trial^18^, liver enzyme abnormalities were reported in 3.8% of AGIs-treated patients (21 of 550 patients), which is similar to the estimated percentage of AGIs-induced liver injury in our study; however the percentage in our study was higher than that of the placebo group in the trial (0.9%, 5 of 567 patients). Numerous clinical trials have reported that patients who receive AGIs therapy are more likely to experience elevation of liver enzymes than those who receive a placebo^2^,^3^, although most of them are asymptomatic and recovered rapidly after discontinuation of drug use^19^. Several early case reports have reported that liver injury typically develops 1–2 months after AGIs therapy is started and has a hepatocellular pattern, with mainly ALT elevation^4^,^5^,^19^. Although this nondeleterious hepatic effect of AGIs during recent or formal use may be a spurious association caused by confounding by contraindication, our findings still support the safety of AGIs use in diabetic patients with advanced CKD in real-world practice. This study also found male as a risk factor for AGIs related liver injury. Drug-induced liver injury (DILI) has been extensively studied and there is still mixed evidence to support the host factors such as sex or chronic liver disease in the development of DILI^20^. Further prospective studies may be warranted to elucidate the host factor including genetic predisposition to the development of AGIs related liver injury.

Glucose control in CKD patients is a complex issue. Progressive loss of renal function impairs renal gluconeogenesis and causes hypoglycemia; conversely, insulin resistance can be worsened by deteriorated renal function and accumulation of uremic toxins, thus causing hyperglycemia^21^. OHA administration complicates this issue further because a reduced GFR leads to the accumulation of these drugs and their active metabolites with a consequent increased risk of side effects^22^. To eliminate the confounding effect of other OHAs on liver injury, subgroup analysis was performed with each OHAs. It proved that whether patients were prescribed with other OHAs or not, there is no increased risk of hepatotoxicity in AGIs group. AGIs have an advantage over other OHAs because they are poorly absorbed into the systemic circulation, and AGIs mechanisms do not expose patients to the risk of hypoglycemia.

The exact mechanisms underlying AGIs-induced liver injury are not well known. In rats, AGIs induced a significant, dose-dependent increase in hepatic glycogen concentrations, which was present after 3, 7, and 28 d of AGIs administration. Light and electron microscopy results proved that the increase in hepatic glycogen concentration was caused by lysosomal storage of glycogen^23^. AGIs are metabolized exclusively and absorbed poorly within the gastrointestinal tract; therefore, systemic toxicity with liver injury is not anticipated and remains unexplained. Early studies on liver biopsy in AGIs-induced hepatitis patients have reported inflammatory cells infiltration in lobules and portal tracts^24^. Animal studies have reported that administration of a high AGIs dose in rats induces lysosomal glycogen storage in the liver^23^. Idiosyncratic reaction-related liver injury is favored over direct toxic reaction-related liver injury, which may be associated with an immunological reaction with the bacterially derived oligosaccharide molecule. Collectively, our results did not indicate the apparent dose-dependent effects of AGIs on liver injury, which is consistent with the proposed idiosyncratic mechanism. We further examined the potentially interactive comorbidities on liver failure, such as old age, concomitant systemic diseases, and chronic liver disease. Predisposing liver disease is theoretically complicated by liver-related alterations in drug metabolism and the possible increased risk of hepatotoxicity. In our study, the risk of AGIs-induced liver injury was not significantly augmented despite disease stratification. Besides, a model excluding previous chronic liver disease, virus hepatitis and liver cirrhosis was performed to consolidate the results. This finding is consistent with that of a previous study, in which AGIs were reported to be a safe and effective drug used in the treatment of cirrhotic patients with low-grade hepatic encephalopathy and type 2 diabetes mellitus^25^.

The strength of our study is the large sample size and nearly complete nationwide coverage of prescriptions and outcomes. The propensity score matching and the nationwide-based study design counteract the selection bias. Using the NHIRD, it is possible to detect the considerably low incidence rate of the adverse effects of drugs. We did not enroll highly selected homogeneous patients, like those recruited in clinical trials. In our study, adverse AGIs effects can be investigated in a real clinical situation. However, there were several limitations. First, drug-induced liver injury was diagnosed by exclusion clinically, and careful evaluations for competing etiologies are essential. However, this was not easily to be achieved with current study design. To minimize the uncertainty of causality between AGIs and liver injury, we further analyzed the DDD influence on outcomes. In addition, we performed multivariate stratified analysis to confirm the robustness of results. Second, the misclassification or unawareness of liver injury by physicians is a probable concern in a register-based study. Liver injury was validated from a local study including randomly sampling records of 102 hospitalized patients in a medical center. The rate of liver injury in our study was similar to previous studies. Third, because the biochemical data, virological profiles, sonographic data, and the response to rechallenge are not available in the Taiwanese NHI database, we were unable to calculate the Council for International Organizations of Medical Sciences scale for causality assessment in drug-induced liver injury^26^ or follow the international drug induced liver injury Expert Working Group consensus^27^. Therefore further prospective studies were needed to elucidate the impact of host factors including sex or genetic predisposition and other socioeconomic factors on the risk of AGIs related liver injury.

In conclusion, we observed that AGIs treatment are not associated with an increased risk of liver injury in advanced CKD patients, even in patients who eventually developed ESRD, and in recent or former AGIs users. Neither accumulated dose-dependent effects of AGIs nor comorbidities of chronic liver disease aggravated AGIs-induced liver injury. Additional randomized controlled trials are warranted to confirm our results.

## Research Design and Methods

### Data source

This was a nationwide population-based cohort study that used data from the National Health Insurance Research Database (NHIRD). The NHIRD contains data released for research purposes in 1999 and randomly selected from the claims records of the National Health Insurance (NHI) program of Taiwan, which is a single-payer system covering nearly 99% of all Taiwanese residents. The database includes all of the registry and claims data, ranging from demographic data to detailed prescribed and dispensed medications in outpatient and inpatient care. The dispensed medications, including names of drugs, were based on the Anatomical Therapeutic Chemical (ATC) classification^28^. The classification includes the quantity, frequency, and dates of drug dispensation and reimbursement. AGIs and other OHAs were identified by their specific ATC code. The NHI claims data regarding medications are reliable because they were collected on the basis of NHI procedures and drug codes that were related to the NHI reimbursement system and were audited^29^,^30^,^31^,^32^,^33^,^34^. The identification numbers of all patients in the databases were encrypted to protect their privacy; therefore, this study was exempted from a full ethical review by the Institutional Review Board of National Taiwan University Hospital (201212021RINC).

### Study subjects

Based on the reimbursement regulations of the NHI, ESA (erythropoietin-stimulating agent) can be prescribed only for anemic advanced CKD patients having a hematocrit level of ≤28% and a serum creatinine level of >6 mg/dL (equivalent to an eGFR of less than 15 mL/min/1.73 m^2^) for achieving a hematocrit level of 33–36%. The baseline comorbidities, including CKD, were identified from at least 3 outpatient visits or one inpatient claim within 1 y preceding the first prescription of ESA with the highest positive accuracy. This identification method has been widely validated with high predictive power. In Taiwan, CKD diagnosis was based on the eGFR according to the The Kidney Disease Improving Global Outcomes (KDIGO) guidelines^35^. A Taiwanese study reported that 85% of patients with advanced CKD not yet requiring dialysis received ESA therapy in 2012^14^,^30^,^33^,^36^. Besides, the median hematocrit level in patients who initiated dialysis was 24.2% in Taiwan. Therefore, this selected cohort is the most representative population of the predialysis stage 4–5 of CKD in Taiwan.

**Figure 3:**
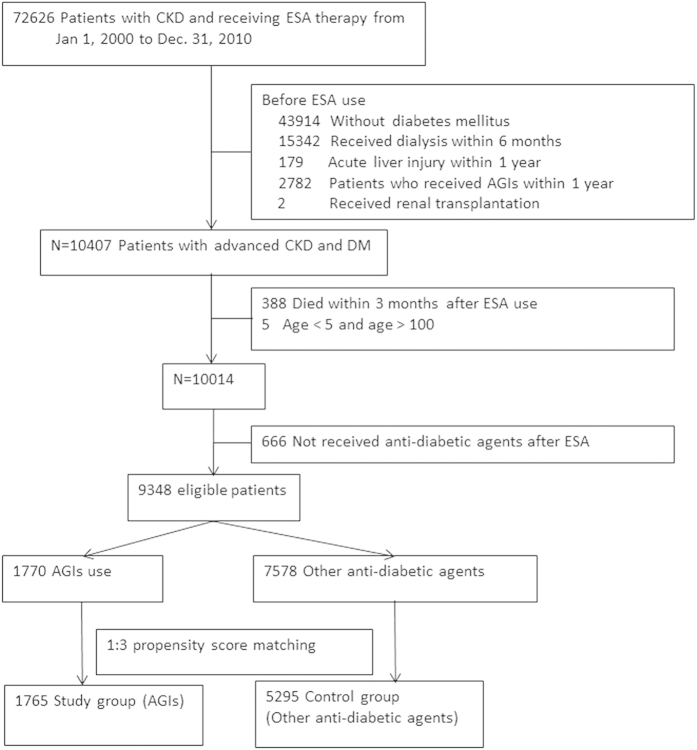
Flow chart presenting the study subjects.

Only patients who did not undergo dialysis (identified using the procedure codes) before and 90 d after the first ESA prescription were enrolled to ensure a sufficient dialysis-free follow-up duration. Patients who underwent renal transplantation before ESA and those who did not survive 90 d after the first ESA treatment were excluded. In addition, we excluded the following patients: age older than 100 y or younger than 20 y, no diagnosis of diabetes mellitus within 1 y, who received AGIs within 1 y, who received renal transplantation, who died within 90 d, and who had a history of acute liver injury within 1 y before the index date (Fig. 3).

The day of ESA prescription was defined as the index date. We extracted all the relevant data, including demographic information, diagnosis codes, and drug prescriptions. Patients who received AGIs therapy after the index date were defined as the study group, and those who did not receive AGIs therapy were matched using the propensity score and defined as the control group. For each patient in the study group, 3 propensity score-matched counterparts were selected from the control group.

### Comorbidities identification

We defined the diseases on the basis of the diagnosis codes of the International Classification of Disease, Ninth Revision, Clinical Modification (ICD-9-CM). The accuracy of the NHIRD claims data for diabetes diagnosis has been validated^14^,^30^,^31^,^33^,^36^,. To balance the risk of diabetes-related severity, we estimated the Diabetes Complications Severity Index (DCSI)^37^ for matching the 2 groups with similar diabetes-related comorbidities.

The ICD-9-CM diagnostic codes defined chronic liver disease, including viral hepatitis, hepatitis B, hepatitis C, and alcoholic liver disease, and other causes of liver cirrhosis (Supplement Table 1). A previous validation study used the hospital administrative database and reported a positive predictive value of 90% on the basis of the aforementioned definition^38^.

### The cumulated dosage of AGIs

For comparing the quantity of drug use to the risk of liver injury, we used the defined daily dose (DDD), which is defined as the assumed average maintenance dose per day for a drug used for its main indication in adults^39^,^40^. According to the WHO definition, 300 mg of acarbose equals 1 DDD. Dose-dependent effects of AGIs on liver injury were examined by analyzing the DDD effects on the risk of liver injury. We defined recent and former AGIs users as patients who filled their prescription for AGIs within 30–60 d and 30–210 d, in that order, before the outcome event date. We did not include medications prescribed within 30 d preceding the outcome event to reduce the potential confounding effects caused by indication.

### Outcomes

The outcome of interest was defined as patients being diagnosed for hepatic injuries, which was mainly adopted from a previous study, carried out by Jinjuvadia *et al.*^41^ and Kao WY *et al.*^42^. The liver injury included disorders of bilirubin excretion (ICD-9-CM code 277.4), acute and sub-acute necrosis of the liver (ICD-9-CM code 570), other sequelae of chronic liver disease (ICD-9-CM code 572.8), hepatitis (ICD-9-CM code 573.3), other specified liver disorders (ICD-9-CM code 573.8), other specified disorders of the biliary tract (ICD-9-CM code 576.8), and jaundice (ICD-9-CM code 782.4). The observation period lasted from the index date to the date of reporting liver injury, to death, or until December 31, 2010, whichever occurred first. In Taiwan, the records were validated for drug-induced liver injury on the basis of a 2-fold increase in the serum ALT level compared with the baseline^43^, and the records were highly accurate with a positive predictive value of 89.2%^43^,^44^. Liver injury was validated using randomly sampling records of 102 hospitalized patients in a medical center.

### Statistical analysis

The demographic characteristics between the study and control groups were compared using a 2-sided *t* test and an X^2^ test. A time-variable cox proportional hazards model was employed to clearly characterize the variability of prescription and the time-course relationship between AGIs use and outcome development. To examine the relationship between the AGIs and liver injury in patients with an underlying liver disease, we further performed multiple stratified analyses. Time-fixed and time-varying analyses were conducted^30^,^33^,^40^.

We evaluated the risk factors of long-term outcomes using a Cox proportional hazards model. To eliminate the confounding factors of underlying liver disease on liver injury, we conducted another sensitivity analysis by excluding patients with chronic liver disease, virus hepatitis and liver cirrhosis^45^. Further, we performed a multilevel discrete-time event history analysis to determine the threshold values of the AGIs dosage for liver injury, using the logistic regression method by incorporating patient-specific random effects and adopting a generalized additive model (GAM) with splines regarding AGIs dosage^40^,^46^. All calculations were performed using R software, Version 2.14.1 (Free Software Foundation, Boston, MA, USA).

## Additional Information

**How to cite this article**: Kao, C.-C. *et al.* Risk of liver injury after α-glucosidase inhibitor therapy in advanced chronic kidney disease patients. *Sci. Rep.* **6**, 18996; doi: 10.1038/srep18996 (2016).

## Supplementary Material

Supplementary Information

## Figures and Tables

**Table 1:** Distribution of characteristics among patients in the study and propensity score-matched control groups.

	Control (n = 5295)	Study (n = 1765)	*P* value
Patient characteristics			
Male	2717 (51.3%)	898 (50.9%)	0.762
Age (y)	63 ± 11	63 ± 11	0.910
Comorbidities			
Charlson score	3.58 ± 1.44	3.57 ± 1.53	0.686
DCSI score	4.63 ± 3.29	4.50 ± 3.24	0.181
Myocardial infarction	146 (2.8%)	37 (2.1%)	0.142
Congestive heart failure	785 (14.8%)	248 (14.1%)	0.437
Peripheral vascular disease	38 (0.7%)	11 (0.6%)	0.744
Cerebrovascular disease	320 (6%)	117 (6.6%)	0.392
Dementia	62 (1.2%)	20 (1.1%)	0.999
COPD	306 (5.8%)	102 (5.8%)	0.999
Rheumatologic disease	21 (0.4%)	7 (0.4%)	0.999
Peptic ulcer	727 (13.7%)	266 (15.1%)	0.166
Hemiplegia	23 (0.4%)	6 (0.3%)	0.674
Tumor	131 (2.5%)	53 (3%)	0.228
Diabetes Mellitus	5225 (98.7%)	1738 (98.5%)	0.555
Chronic liver disease	170 (3.2%)	63 (3.6%)	0.489
Liver cirrhosis	105 (2%)	38 (2.2%)	0.696
ALD	15 (0.3%)	5 (0.3%)	0.999
HBV carrier	50 (0.9%)	15 (0.8%)	0.776
HCV carrier	58 (1.1%)	21 (1.2%)	0.794
Time-varying			
ESRD	4368 (82.5%)	1629 (92.3%)	<0.001
Drug			
Insulin (short- and intermediate-acting)	4191 (79.2%)	1498 (84.9%)	<0.001
Insulin (long-acting)	862 (16.3%)	500 (28.3%)	<0.001
Biguanide	472 (8.9%)	312 (17.7%)	<0.001
DPP-4 inhibitor	623 (11.8%)	407 (23.1%)	<0.001
Meglitinide	2411 (45.5%)	1216 (68.9%)	<0.001
Thiazolidinedione	866 (16.4%)	701 (39.7%)	<0.001
Sulfonylurea	2475 (46.7%)	1300 (73.7%)	<0.001
Outcome Liver injury	173 (3.3%)	68 (3.9%)	0.256

Descriptive statistics for categorical variables were expressed as frequency and percentage, while continuous variables were expressed as mean ± standard deviation as appropriate.

ALD, alcoholic liver disease; COPD, chronic obstructive pulmonary disease; DCSI, Diabetes Complications Severity Index; DPP-4 inhibitor: dipeptidyl peptidase-4 inhibitor; ESRD, end-stage renal disease.

**Table 2:** Risk of liver injury following AGIs treatment according to the time-varying Cox regression model.

Covariate	Hazard Ratio (95% CI)	*P* value
Male	1.33 (1.03–1.72)	0.028
Chronic liver disease	2.08 (1.17–3.70)	0.012
AGIs	1.56 (0.80–3.04)	0.190
Mean DDD ^a^ within 30–60 d	2.92 (0.27–31.48)	0.376
Mean DDD within 30–210 d	0.39 (0.04–4.31)	0.442

CI, confidence interval; DDD, defined daily dose.

a: Defined daily dose (DDD), which is defined as the assumed average maintenance dose per day for a drug used for its main indication in adults. According to the WHO definition, 300 mg of acarbose equals 1 DDD.
