# Supplementary material for: Risk of liver injury after α-glucosidase inhibitor therapy in advanced chronic kidney disease patients
Source: Sci Rep. 2016 Jan 11;6:18996. doi: 10.1038/srep18996 (PMC4707434; doi:10.1038/srep18996)
Supplement: Supplementary Information [file srep18996-s1.doc]

**Risk of liver injury after α-glucosidase inhibitor therapy in advanced chronic kidney disease patients**

Chih-Chin Kao^1, 2^, Pei-Chen Wu^3^, Che-Hsiung Wu^4^, Li-kwang Chen^5^, Hsi-Hsien Chen^1,6^, Mai-Szu Wu^1,6^ ,Vin-Cent Wu^7^

^1^Division of Nephrology, Department of Internal Medicine, Taipei Medical University Hospital, Taipei, Taiwan

^2^Graduate Institute of Clinical Medicine, College of Medicine, Taipei Medical University, Taipei, Taiwan

^3^Division of Nephrology, Department of Internal Medicine, Mackay Memorial Hospital, Taipei, Taiwan

^4^Division of Nephrology, Taipei Buddhist Tzu Chi General Hospital, Buddhist Tzu Chi University, Taipei, Taiwan

^5^Institute of Population Health Sciences, National Health Research Institutes, Zhunan, Taiwan

^6^Department of Internal Medicine, School of Medicine, Taipei Medical University, Taipei, Taiwan

^7^Department of Internal Medicine, National Taiwan University Hospital, Taipei, Taiwan

**Supplementary Table 1:** ICD-9-CM diagnostic codes

| **Outcome** | **ICD9-codes** |
| --- | --- |
| **Acute Hepatitis** | 277.4, 570, 572.8, 573.3, 573.8, 576.8, 782.4 |

**Supplement table 2.** Risk of liver injury following AGIs treatment according to the time-varying Cox regression model (excluding chronic liver disease, virus hepatitis and liver cirrhosis patients)

| **Covariate** | **Hazard Ratio (95% CI)** | ***P* value** |
| --- | --- | --- |
| **Male** | 1.32 (1.01–1.72) | 0.039 |
| **AGIs** | 1.49 (0.75–2.98) | 0.255 |
| **Mean DDD ^a^ within 30–60 d** | 2.97 (0.26–33.70) | 0.380 |
| **Mean DDD within 30–210 d** | 0.53 (0.04–5.44) | 0.532 |

CI, confidence interval; DDD, defined daily dose

a: Defined daily dose (DDD), which is defined as the assumed average maintenance dose per day for a drug used for its main indication in adults. According to the WHO definition, 300 mg of acarbose equals 1 DDD.
